# Ho:YAG laser at 2097 nm pumped by a narrow linewidth tunable 1.91 μm laser

**DOI:** 10.1038/s41598-023-27970-0

**Published:** 2023-01-18

**Authors:** Juntao Tian, Lili Zhao, Zhiyong Li, Jintian Bian, Qing Ye, Hai Wang, Rongqing Tan

**Affiliations:** 1 Present Address: Laser Engineering Center, Aerospace Information Research Institute, Chinese Academy of Sciences, Beijing, 100094 China; 2 School of Electronic, Electrical and Communication Engineering, University of Chinese Academy of Sciences, Beijing, 100049 China; 3 State Key Laboratory of Pulsed Power Laser Technology, College of Electronic Countermeasures, National University of Defense Technology, Hefei, 230037 China; 4 Anhui Laboratory of Advanced Laser Technology, College of Electronic Countermeasures, National University of Defense Technology, Hefei, 230037 China

**Keywords:** Lasers, LEDs and light sources, Solid-state lasers

## Abstract

This study presents a high efficiency Ho:YAG laser based on a narrow linewidth tunable 1.91 μm laser. A tunable Tm:YLF laser is the pump source and the wavelength continuous tunability ranges from 1906.04 to 1908.83 nm, corresponding to a linewidth of less than 0.41 nm. The tunable Tm:YLF laser is achieved by changing the operating temperature of the VBG. The output power of the Ho:YAG laser is between 21.04 and 23.53 W and the slope efficiency is between 64.08 and 68.26% at the pump power of 39.8 W. The output power and slope efficiency corresponding to the pump wavelength of 1907.36 nm are 23.53 W and 68.26%, respectively. This study illustrates that fine-tuning the pump wavelength is an effective way to improve the slope efficiency and output power of the Ho:YAG laser at room temperature.

## Introduction

A 2.1 μm laser is located in the atmospheric window and widely used in applications such as laser medical^1^, material processing^2^, optical communication lidar detection and ranging systems^3^, and the optical parametric oscillators^4,5^. At present, the main approach to obtain lasers with wavelengths at 2.1 μm is to pump a Ho-doped crystal with a 1.9 μm laser that coincides with the absorption wavelength peak of Ho-doped crystal. A solid-state laser pumped by a 1.9 µm laser can achieve a 2.1 µm laser with high power, high beam quality, and narrow linewidth at room temperature. Until now, the Tm-doped solid-state and Tm-doped fiber lasers were used as the pump sources in a high power Ho:YAG laser owing to the better beam quality and higher power. Shen et al. reported a Ho:YAG laser pumped with a Tm:YLF laser at 1907.8 nm, and the output power was 103 W with a slope efficiency of 67.8%^6^. Pumping the Ho:YAG laser with a Tm-doped fiber laser is a more compact approach due to qualities such as simpler thermal management, higher conversion efficiency, and smaller volume. An output power of 36 W for the Ho:YAG laser pumped at 1907 nm by Tm-doped fibre laser has been reported by Antipov et al.^7^.

Using a narrow linewidth pump source is an effective way to increase the target laser efficiency^8,9^. The central wavelength and linewidth of a pump laser have a great influence on the Ho:YAG laser due to the narrow absorption wavelength peak of Ho:YAG crystal at 1.9 μm. Therefore, some research on the Ho:YAG laser has concentrated on narrow linewidth pump sources. Currently, lasers using volume Bragg grating (VBG) have the ability to select wavelength and narrow linewidth. Chen et al. reported a Tm:YLF laser with a VBG, and the output power was 15.5 W at 1908.1 nm, corresponding to the linewidth of 0.15 nm^10^. Wei et al. reported a narrow linewidth Tm:YLF laser with a VBG, and the output power was 202 W at 1908.5 nm, corresponding to the linewidth of 0.57 nm^11^. At present, there are few reports about tunable Tm:YLF lasers. Sheintop et al. reported a tunable Tm:YLF laser, corresponding to a tunable range of 1926–1961 nm^12^. Here, the absorption cross-section of the Ho:YAG crystal at 1.91 μm was higher than that at 1.93 μm, which assisted in the absorption of the pump light and achieved higher output power^13^. Therefore, a Ho:YAG laser pumped by a tunable 1.91 μm laser is an attractive method to obtain higher slope efficiency and output power.

This study proposes a Ho:YAG laser pumped by a narrow linewidth tunable 1.91 μm laser. The pump source was a Tm:YLF laser with a tunable range of 1906.04–1908.83 nm, corresponding to a linewidth of less than 0.41 nm. The beam quality of the Tm:YLF laser remains almost constant over the entire tuning range, which was favorable for pumping the Ho:YAG laser. The output characteristics of the Ho:YAG laser at different pump wavelengths are measured.

## Experimental setup

**Figure 1:**
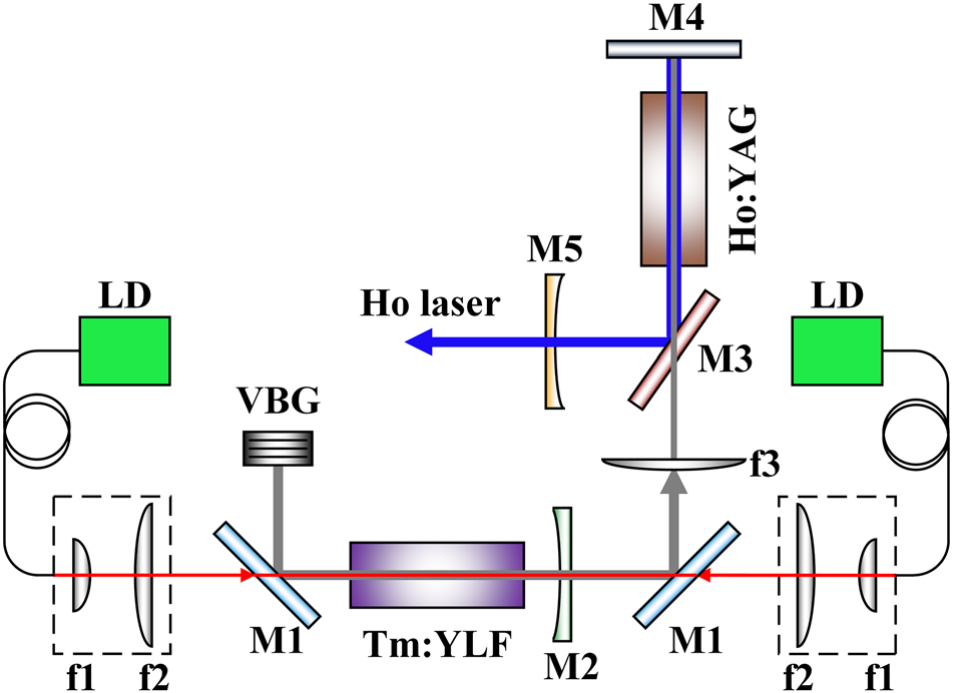
Experimental setup of the Ho:YAG laser pumped by Tm:YLF laser.

The experimental setup of the Ho:YAG laser pumped by Tm:YLF is shown in Fig. 1.

A double-ended pump structure was used for the Tm:YLF laser. The pump sources were two fiber-coupled laser diodes (LDs) with a power of 80 W and central wavelength of 793 nm. The pump beams were focused on the Tm:YLF crystal with a diameter of 0.75 mm using beam shaping devices. The M1s were coated with high transmittivity at 793 nm and high reflection at 1.9 μm. M2 was a plano-concave mirror with a transmittance of 20% at 1.9 μm, and its radius of curvature was 150 mm. The transverse size and thickness of the VBG were 4 × 3 mm^2^ and 5 mm, respectively. The diffraction efficiency was greater than 99.0% at 1905.5 nm at room temperature. The structure was mounted on a copper heat sink that could be controlled from room temperature to 210 °C. The entire cavity consisted of a plano-concave mirror M2, 45° dichroic mirror M1, and VBG. The cavity length of the Tm:YLF laser was 85 mm in this experiment. The Tm:YLF crystal was a-cut with a size of 1.5 × 4 × 30 mm^3^ and doping concentration of 2.0 at.%. Both ends of the Tm:YLF crystal were coated with antireflection coatings of 793 nm and 1.9 μm, respectively. Additionally, the crystal was wrapped using an indium foil and fastened into a copper holder, which was water cooled to 20 °C.

A single-end-pump structure was used for Ho:YAG laser. The pump lasers were focused into the Ho:YAG crystal with a beam diameter of approximately 0.95 mm by lens f3. M3 was a 45° dichroic mirror with high transmittivity at 1.9 μm and high reflection at 2.1 μm. M4 was coated with high transmittivity at 1.9 μm and high reflection at 2.1 μm. M5 was a plano-concave mirror with a transmittance of 20% at 2.1 μm, and the radius of curvature was 300 mm. Furthermore, the entire cavity consisted of a plano-concave mirror M5, 45° dichroic mirror M3, and plane mirror M4. The cavity length of the Ho:YAG laser was 115 mm. The Ho:YAG crystal had a diameter of 4 mm, length of 50 mm, and doping concentration of 0.8 at.%. The two end surfaces of the crystal were anti-reflection (R ≤ 0.3%) coated at 1.9 and 2.1 μm. Lastly, the crystal was wrapped in a copper holder filled with flowing water and the temperature was controlled at 20 °C.

## Results and discussion

**Figure 2:**
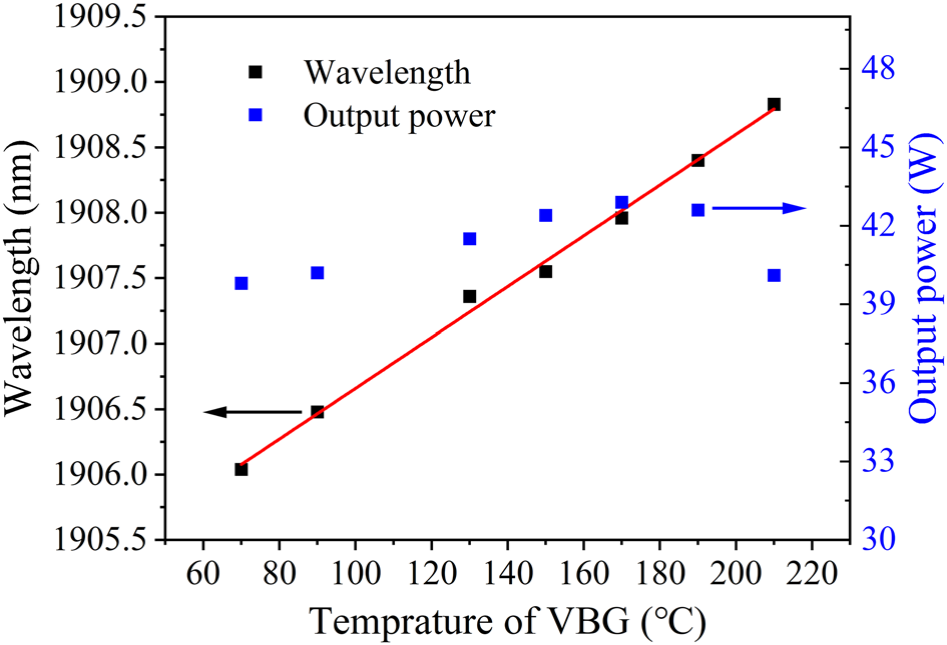
Wavelengths at different VBG temperature.

The wavelengths of the Tm:YLF laser were measured in the experiment by a Fourier transform infrared spectrometer (Nicolet iS50 FTIR), as shown in Fig. 2. The wavelength of the Tm:YLF laser varied with the operating temperature of the VBG. The wavelength increased from 1906.04 to 1908.83 nm, and the corresponding VBG operating temperature increased from 70 to 210 °C at the output power of 39.8 W. The wavelength tuning range was 2.79 nm.

The output powers of the Tm:YLF laser were measured at different VBG temperature. The initial operating temperature of the VBG was set to 70 °C and high output power experiment was not carried out at 110 °C to avoid crystal damage due to the water absorption wavelength in the near-infrared band. The output powers of the Tm:YLF laser at different VBG temperature and at pump power of 105.5 W were shown in Fig. 2. The minimum and maximum output powers in the entire tuning range were 39.8 and 42.9 W, and the corresponding output wavelengths were 1906.04 and 1907.96 nm, respectively.

**Figure 3:**
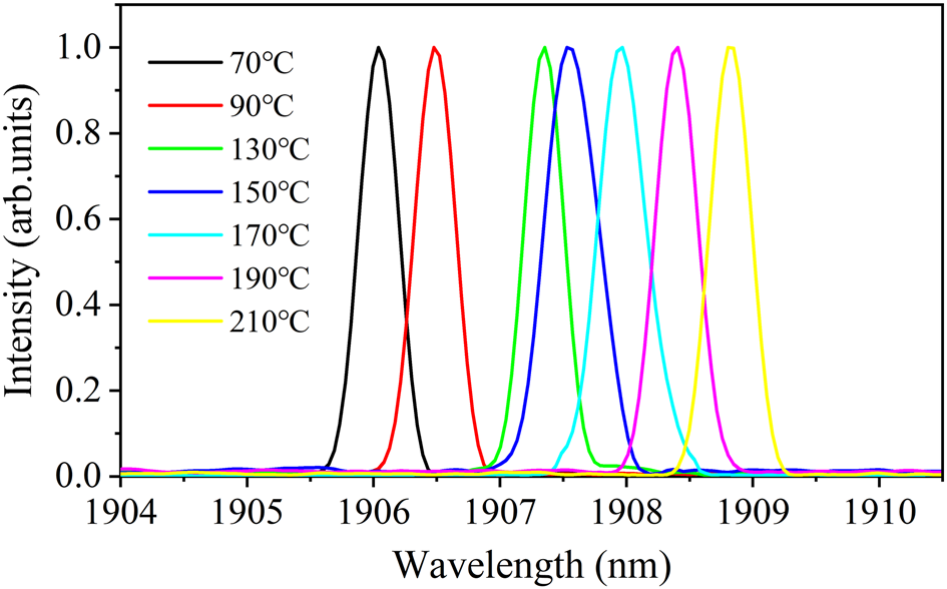
Spectra of the Tm:YLF laser.

The linewidths of the Tm:YLF laser at different VBG temperature are shown in Fig. 3.

The color curves represent the Tm:YLF laser output spectra at different VBG temperature of 70 °C, 90 °C, 130 °C, 150 °C, 170 °C, 190 °C, and 210 °C; where the center wavelengths were 1906.04, 1906.48, 1907.36, 1907.55, 1907.96, 1908.40, and 1908.83 nm, respectively. The corresponding linewidths were 0.39, 0.38, 0.35, 0.41, 0.34, 0.34, and 0.35 nm. The Tm:YLF laser exhibited narrow linewidth and high stability, and the linewidths were between 0.34 and 0.41 nm in the entire tuning range.

**Figure 4:**
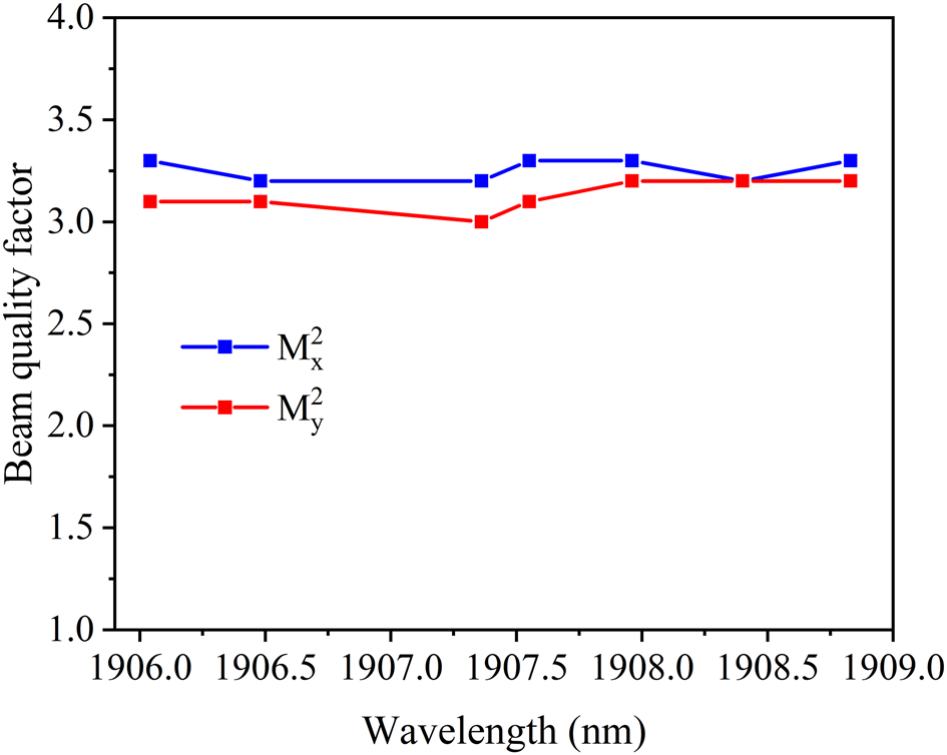
Beam quality factor of Tm:YLF laser.

The 10/90 knife edge technology was used to measure the beam radius of the Tm:YLF laser, and the beam quality factor was calculated using Gaussian fitting. The beam quality factors in the horizontal and vertical directions at different output wavelengths were approximately 3.3 and 3.1, respectively, as shown in Fig. 4.

The beam quality remained almost constant over the entire tuning range, which was favorable for pumping the Ho:YAG laser. Therefore, a narrow linewidth 1.91 μm source was obtained with a stable beam quality, tuning range of 1906.04–1908.83 nm, and output power of 39.8 W.

**Figure 5:**
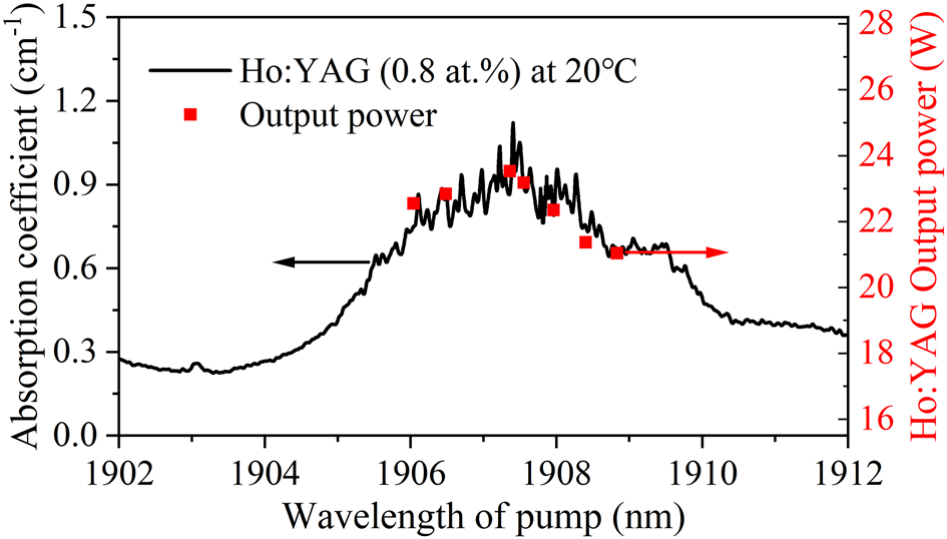
Absorption spectrum of 0.8 at.% doped Ho:YAG at 20 °C and output power.

The measured absorption spectrum of the Ho:YAG crystal in the 1902–1912 nm range at room temperature (20 °C) is represented by the black line in Fig. 5. The red squares represent the maximum output powers of the Ho:YAG laser corresponding to different pump wavelengths under the pump power of 39.8 W. The output powers aligned much better with the Ho:YAG absorption spectrum in the tuning range of 1906.04–1908.83 nm. The results demonstrated that the transmittance at different pump wavelengths had an effect on the Ho:YAG laser output power.

The output characteristics of the Ho:YAG laser versus the tunable pump wavelength are shown in Table 1. By comparing the output power of Ho:YAG laser at different pump wavelengths, it was found that the output power was at 21.04–23.53 W and the corresponding slope efficiency was at 64.08–68.26%. In addition, the conversion efficiency was between 52.86 and 59.12% over the entire tuning range. Additionally, the laser had high conversion efficiency at different pump wavelengths, and the higher power of Ho:YAG laser could be obtained by fine-tuning the pump wavelength.

**Table 1.** Output characteristics of Ho:YAG laser.

Pump wavelength (nm)	Output power (W)	Slope efficiency (%)	Conversion efficiency (%)
1906.04	22.55	66.70	56.66
1906.48	22.84	68.08	57.39
1907.36	23.53	68.26	59.12
1907.55	23.18	68.01	58.24
1907.96	22.35	66.10	56.16
1908.40	21.37	64.24	53.69
1908.83	21.04	64.08	52.86

**Figure 6:**
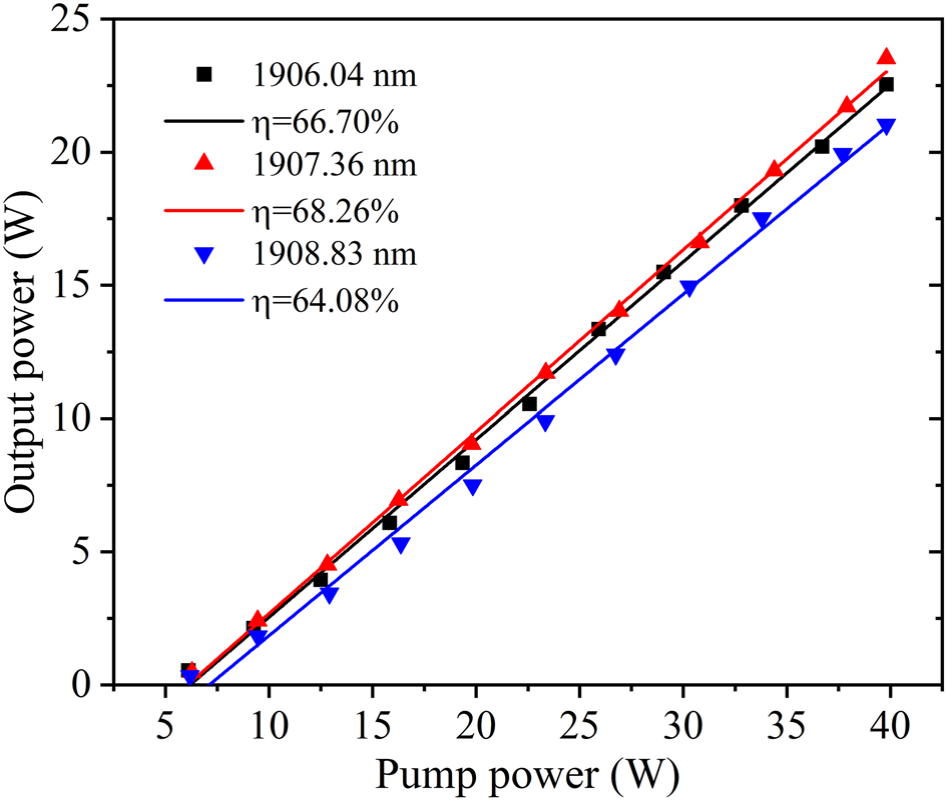
The output powers of Ho:YAG laser.

The output power of Ho:YAG laser versus tunable pump power were shown in Fig. 6. The pump power was 39.8 W at different wavelengths of 1906.04, 1907.36, and 1908.83 nm; here, the maximum output powers of the Ho:YAG laser were 22.55, 23.53, and 21.04 W, respectively. The corresponding slope efficiencies η were 66.70%, 68.26%, and 64.08%, and the corresponding conversion efficiencies were 56.66%, 59.12%, and 52.86%. There is no absorption saturation phenomenon at different pump wavelengths.

**Figure 7:**
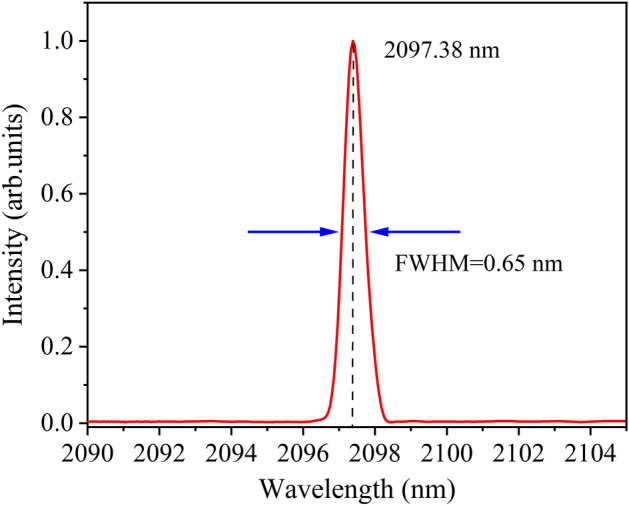
Spectrum of the Ho:YAG laser.

The spectrum of the Ho:YAG laser was measured using a Fourier transform infrared spectrometer (Nicolet iS50 FTIR). The full width at half maximum (FWHM) was 0.65 nm and the wavelength peak at 2097.38 nm, as shown in Fig. 7.

**Figure 8:**
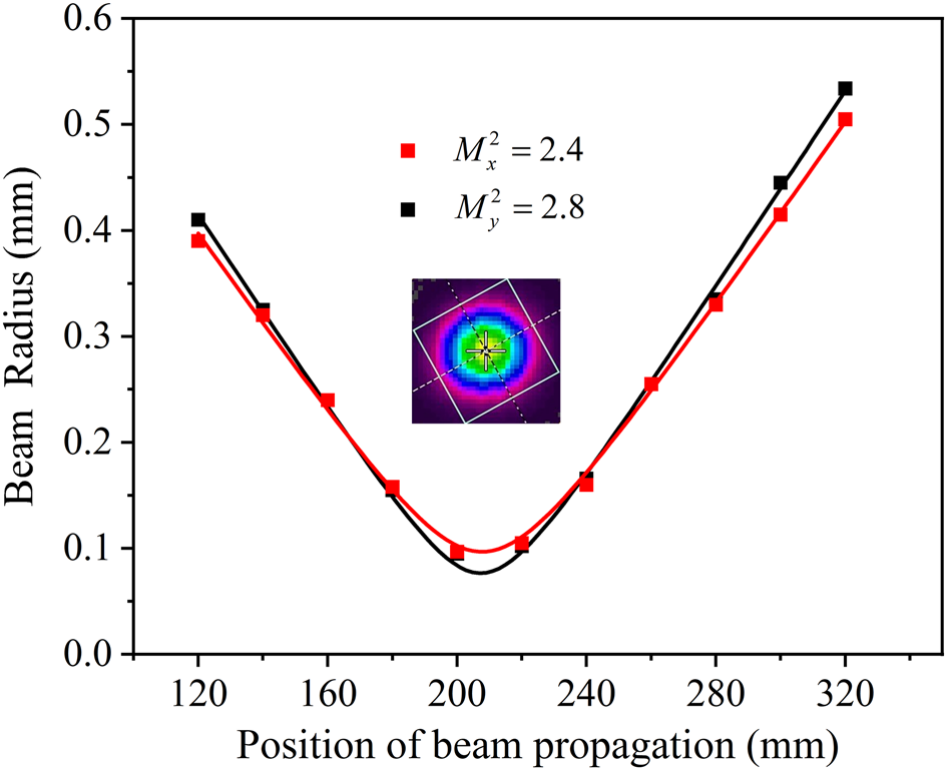
Beam quality factor measurement of Ho:YAG laser.

The beam radius was measured at an output power of 23.53 W using 90/10 knife edge technology. The beam quality factor M2 was calculated with Gaussian fitting, as shown in Fig. 8. The beam quality factors in the horizontal and vertical directions were 2.4 and 2.8, respectively. The inset in Fig. 8 shows the transverse beam profile recorded by a pyroelectric camera (Pyrocam III, Spiricon).

## Conclusions

This study demonstrated a Ho:YAG laser pumped by a narrow linewidth tunable 1.91 μm laser. The pump laser was a Tm:YLF laser with an output power of 39.8 W and a tunable range of 1906.04–1908.83 nm, corresponding to a linewidth of less than 0.41 nm. The beam quality of the Tm:YLF laser remained almost constant over the entire tuning range. The output power of the Ho:YAG laser was at 21.04–23.53 W and the corresponding slope efficiency was at 64.08–68.26%. In addition, the conversion efficiency was between 52.86 and 59.12% over the entire tuning range. The Ho:YAG laser operated stably at 2097.38 nm with a linewidth of 0.65 nm at different pump wavelengths. This study illustrates that the pump wavelength has an effect on the output performance of the Ho:YAG laser in the range of 1906.04–1908.83 nm. At the pump wavelength of 1097.36 nm, the output power and slope efficiency of the Ho:YAG laser reach the maximum of 68.26% and 23.53 W, respectively.

## Data Availability

The datasets used and analysed during the current study available from the corresponding author on reasonable request.
